# Malignant tumor increases the risk of all causes in-hospital mortality in critically ill patients with ischemic stroke: analysis of the MIMIC-IV database

**DOI:** 10.1186/s12883-024-03690-4

**Published:** 2024-05-27

**Authors:** Qiwei Wang, Wang Fu, Feng Wang

**Affiliations:** Department of Neurology, Seventh People’s Hospital of Shanghai University of Traditional Chinese Medicine, Shanghai, P.R. China

**Keywords:** Ischemic stroke, Malignant tumor, In-hospital mortality, ICU, MIMIC database

## Abstract

**Background:**

Ischemic stroke (IS) and malignant tumor (MT) have high morbidity and mortality rates worldwide, and several associations exist between them. This study aimed to determine the effect of MT on hospital mortality in patients with IS.

**Methods:**

Based on their MT status, participants with IS in the Medical Information Mart for Intensive Care IV (MIMIC-IV) were divided into two groups. The primary outcome was in-hospital all causes mortality. Kaplan-Meier survival analysis was performed to evaluate the intergroup in-hospital mortality, and three Cox regression models were used to determine the association between MT and in-hospital mortality.

**Results:**

A total of 1605 participants (749 males and 856 females) were included in the study. The mean age was 72.030 ± 15.463 years. Of these, 257 (16%) patients died in the hospital. Kaplan-Meier analysis showed that the MT group had a significantly lower possibility of in-hospital survival than the non-MT group. In the unadjusted model, in-hospital mortality among MT patients had a higher odds ratio (OR) of 1.905 (95% CI, 1.320–2.748; *P* < 0.001) than the non-MT group. After adjusting for basic information, vital signs, and laboratory data, MT was also associated with increased in-hospital mortality (OR = 1.844, 95% CI: 1.255–2.708; *P* = 0.002).

**Conclusions:**

Among the patients with IS, the risk of all causes in-hospital mortality was higher for MT than for patients non-MT. This finding can assist clinicians in more accurately assessing prognosis and making informed treatment decisions.

## Introduction

Over 10 million people die of malignant tumors (MT) annually, with 23 million new cases, making MT one of the major causes of death globally [1]. MT is either the first or second leading cause of death before the age of 70 in 112 countries, according to the World Health Organization [2]. Although most MT patients die of cancer, many die of other diseases, including stroke. Stroke has been consistently ranked as one of the leading causes of death worldwide [3]. Moreover, it is anticipated that the prevalence of cancer and stroke will rise concurrently with the aging of the worldwide populace and the escalating average age at the point of mortality [1, 3].

Previous research has indicated a correlation between ischemic stroke (IS) and MT, with one in ten individuals with IS having a concurrent MT [4]. MT has the potential to influence the pathophysiology of stroke either through direct mechanisms or by contributing to coagulation disorders, leading to a state of hypercoagulation. Additionally, the risk of stroke may be exacerbated by infections associated with MT, such as lung cancer-related lung infections. Furthermore, various MT treatment modalities, including chemotherapy, radiotherapy, and surgery, have been demonstrated to elevate the likelihood of stroke [5]. Patients with IS comorbid with MT tend to be more symptomatic at onset and have a poorer prognosis [6]. Furthermore, the rate of fatal stroke in patients with MT is 21.64 per 100,000-person years [7]. An increasing body of evidence suggests that IS is linked to a heightened risk of in-hospital mortality among individuals with MT. However, whether this correlation persists in patients with IS, a population generally presenting with more severe pathophysiological conditions, remains uncertain. Therefore, assessing the impact of MT on mortality in critically ill patients with IS can help identify high-risk populations for medical management or timely treatment to reduce mortality [6, 8].

Consequently, the primary objective of this investigation was to examine the influence of MT on in-hospital mortality among critically ill patients diagnosed with IS, utilizing data from the Medical Information Mart for Intensive Care IV (MIMIC-IV) database for comprehensive analysis.

## Methods

### Data source

The primary data used in this study were obtained from the MIMIC-IV database (version 2.0). The MIMIC-IV database encompasses comprehensive medical records of individuals admitted to the intensive care unit (ICU) or emergency department at Beth Israel Deaconess Medical Center in Boston, Massachusetts, USA, spanning the period from 2008 to 2019 [9]. Our research utilized publicly accessible, de-identified data, and as a result, the requirement for patient consent was waived. All steps were performed according to the relevant guidelines to protect patient privacy. One of the authors (WQ W ID:12,630,989) received authorization to document this database following online training at the National Institutes of Health.

### Patient inclusion and exclusion

The diagnosis of IS was determined according to the International Classification of Diseases, 9th and 10th editions [10]. Inclusion criteria for this study were (I) patients who were admitted for the first time with a diagnosis of IS (*n* = 4375). Exclusion criteria included (I) patients with repeat admissions (*n* = 28), (II) patients who were not admitted to the ICU for treatment (*n* = 2742), and (III) patients younger than 18 years of age at the time of first admission.

### Clinical and laboratory data

MT or non-MT information is our primary independent variable, and non-MT in our study refers to people with benign tumors or no tumors.

We further gathered patients’ basic information, including age, sex, and weight. The presence of comorbidities upon admission was also documented, encompassing conditions such as diabetes, chronic lung disease, myocardial infarction, and heart failure. Simultaneously, we extracted the initial recorded laboratory data and vital signs of patients with IS upon admission. Laboratory data included creatinine, blood urea nitrogen (BUN), anion gap, chloride, glucose, hemoglobin, white blood cell and platelet counts; potassium, sodium, and calcium levels; as well as prothrombin time (PT). Vital signs included systolic blood pressure (SBP), diastolic blood pressure (DBP), mean blood pressure (MBP), body temperature, heart rate, respiratory rate (RR), and pulse oximetry-derived oxygen saturation (SPO2). In addition, Glasgow Coma Scale (GCS) scores and Sequential Organ Failure Assessment (SOFA) scores were collected from each patient, and patients were categorized into a mild to moderate organ failure group and a severe organ failure group according to the SOFA 2 cut-off [11–13]. The endpoint of our study was in-hospital mortality, indicating the survival status at the time of discharge.

### Statistical analysis

Variables with missing data exceeding 20% of the total were excluded, and for variables with less than 20% missing data, we employed multiple imputations to fill in the gaps. This is done by generating five datasets through multiple interpolations, applying statistical models to each complete dataset, and finally integrating the results of the analysis [14–16].

Non-normally distributed continuous variables were presented as medians with upper and lower quartiles; otherwise, they were reported as the mean ± standard deviation (SD). Group comparisons were conducted using the T-test or Wilcoxon rank-sum test for continuous variables, and the Chi-square test, Fisher’s exact test or Yates’ continuity corrected chi-squared test for categorical variables. Hazard ratios (HR) with 95% confidence intervals (CI) were used to express the results. All tests were two-tailed, and a P-value of ≤ 0.05 was considered statistically significant. Statistical analyses were carried out using R version 3.6.3 (R Foundation for Statistical Computing, Vienna, Austria).

## Results

### Patient characteristics

**Fig. 1:**
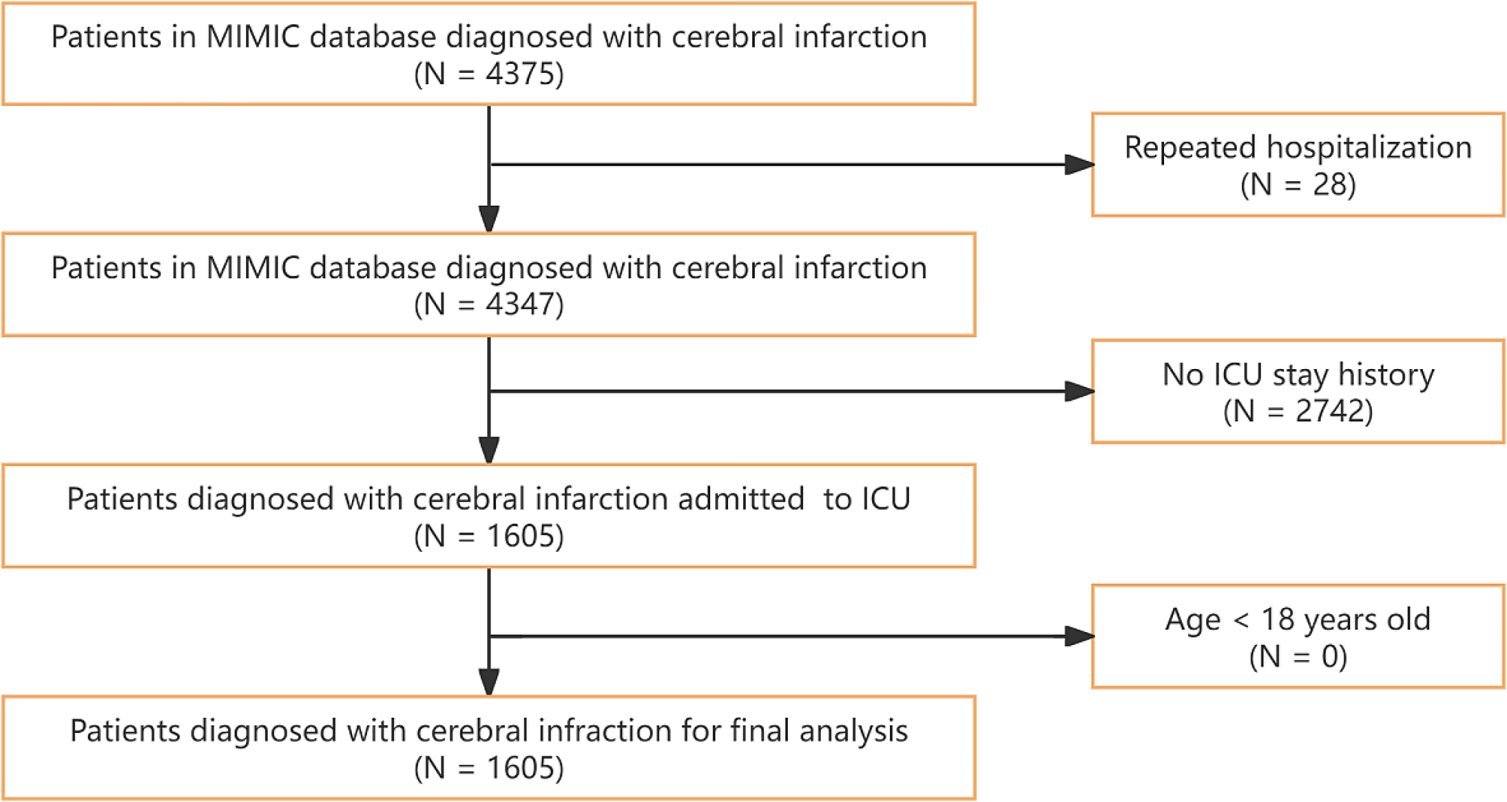
Inclusion and exclusion flowchart of the study

A total of 1605 eligible patients with IS (1493 non-MT and 112 MT patients) were included in the study (Fig. 1). The mean age was 72.030 ± 15.463 years. Out of these, 257 patients (224 non-MT and 33 MT patients) died in the hospital, with an in-hospital mortality rate of 16.0%. According to their cancer history, the patients were divided into two groups. Table 1 provides a summary of the baseline characteristics of the patients. Compared to non-MT, patients with MT were more likely to be male, weigh less, have lower SBP, have higher heart rate, have less comorbid diabetes, have more comorbid myocardial infarction, have lower hemoglobin levels, lower calcium levels, lower sodium levels, and higher SOFA scores on admission (*P* < 0.05).

**Table 1:** Characteristics of included patients

Characteristic	Total (*n* = 1605)	No-cancer (*n* = 1493)	Cancer (*n* = 112)	*P* value
Demographics				
Age, years	72.030 (15.463)	71.950 (15.652)	73.097 (12.687)	0.449
Sex				0.016
Male	749 (46.7)	684 (45.8)	65 (58.0)	
Female	856 (53.3)	809 (54.2)	47 (42.0)	
Ethnicity (%)				0.506
American Indian	2 (0.1)	2 (0.1)	0 (0.0)	
Asian	50 (3.1)	48 (3.2)	2 (1.8)	
Black American	183 (11.4)	173 (11.6)	10 (8.9)	
Others	69 (4.3)	64 (4.3)	5 (4.5)	
Unable to obtain	18 (1.1)	16 (1.1)	2 (1.8)	
Unknown	269 (16.8)	254 (17.0)	15 (13.4)	
White	963 (60.0)	886 (59.3)	77 (68.8)	
Hispanic	51 (3.2)	50 (3.3)	1 (0.9)	
Weight, kg	78.283 (23.528)	78.705 (23.991)	72.654 (15.120)	0.009
SBP, mmHg	136.691 (18.295)	136.984 (18.308)	132.773 (17.747)	0.019
DBP, mmHg	71.939 (12.371)	72.033 (12.478)	70.695 (10.810)	0.270
MBP, mmHg	89.149 (12.296)	89.291 (12.373)	87.248 (11.084)	0.090
Heart rate, BMP	79.857 (14.620)	79.421 (14.529)	85.658 (14.662)	< 0.001
Respiratory rate, BMP	19.247 (3.214)	19.204 (3.216)	19.817 (3.144)	0.052
Temperature, ℃	36.910 (0.421)	36.911 (0.419)	36.898 (0.438)	0.747
SPO2, %	96.968 (2.167)	96.965 (2.172)	97.004 (2.109)	0.853
Comorbidities, n (%)				
Diabetes, n(%)	518 (32.3)	492 (33.0)	26 (23.2)	0.043
Myocardial infarction, n(%)	184 (11.5)	162 (10.9)	22 (19.6)	0.008
Congestive heart failure, n(%)	387 (24.1)	354 (23.7)	33 (29.5)	0.208
Chronic pulmonary disease, n(%)	267 (16.6)	244 (16.3)	23 (20.5)	0.309
Dementia, n(%)	88 (5.5)	83 (5.6)	5 (4.5)	0.783
Rheumatic disease, n(%)	35 (2.2)	32 (2.1)	3 (2.7)	0.969
Peptic ulcer disease, n(%)	15 (0.9)	14 (0.9)	1 (0.9)	1.000
Renal disease, n(%)	280 (17.4)	263 (17.6)	17 (15.2)	0.599
Mild_liver_disease, n(%)	46 (2.9)	45 (3.0)	1 (0.9)	0.315
Severe_liver_disease, n(%)	7 (0.4)	6 (0.4)	1 (0.9)	0.986
AIDS^a^, n(%)	< 10 (< 1)	0 (0.0)	< 10 (< 1)	0.091
Laboratory parameters				
Anion gap, mmol/L	14.500 (13.000, 16.500)	14.500 (13.000, 16.500)	15.000 (13.000, 16.500)	0.747
BUN, mg/dL	21.050 (13.556)	20.974 (13.701)	22.058 (11.440)	0.415
Bicarbonate, mmol/L	23.703 (3.252)	23.683 (3.166)	23.964 (4.237)	0.378
Calcium, mmol/L	8.749 (0.613)	8.763 (0.613)	8.570 (0.594)	0.001
Creatinine, mg/dL	1.142 (1.046)	1.149 (1.074)	1.054 (0.545)	0.351
Chloride, mmol/L	104.023 (5.318)	104.091 (5.334)	103.121 (5.028)	0.063
Glucose, mg/dL	136.776 (52.035)	136.896 (50.526)	135.174 (69.347)	0.736
Hemoglobin, g/dL	12.280 (2.061)	12.361 (2.025)	11.200 (2.243)	< 0.001
Potassium, mmol/L	4.064 (0.681)	4.064 (0.679)	4.074 (0.714)	0.881
Sodium, mmol/L	139.715 (3.876)	139.783 (3.816)	138.804 (4.524)	0.010
PT, sec	13.668 (3.930)	13.660 (3.988)	13.774 (3.079)	0.767
INR	1.245 (0.374)	1.244 (0.380)	1.250 (0.284)	0.869
PTT, sec	33.743 (14.727)	33.786 (14.827)	33.163 (13.357)	0.666
WBC, 10^9^/L	9.800 (7.800, 12.400)	9.800 (7.800, 12.300)	10.100 (7.400, 14.750)	0.170
Platelets, 10^9^/L	220.500 (177.500, 275.500)	220.500 (178.500, 274.000)	225.000 (154.000, 289.750)	0.464
Scoring systems				
SOFA	3.000 (2.000, 5.000)	3.000 (2.000, 5.000)	4.000 (2.000, 6.000)	0.004
GCS	13.000 (9.000, 14.000)	13.000 (9.000, 14.000)	12.000 (8.000, 14.000)	0.312
Outcomes				
ICU LOS, days	2.390 (1.300, 4.780)	2.340 (1.300, 4.780)	2.925 (1.450, 4.822)	0.425
HOS LOS, days	6.524 (3.692, 12.665)	6.431 (3.682, 12.622)	7.459 (4.545, 12.776)	0.225
HOS mortality, n (%)	257 (16.0)	224 (15.0)	33 (29.5)	< 0.001

MT, malignant tumor; SBP, systolic blood pressure; DBP, diastolic blood pressure; MBP, mean blood pressure; SPO2, pulse oximetry-derived oxygen saturation; AIDS, Acquired Immunodeficiency Syndrome; BMP, beat per minute; BUN, blood urea nitrogen; PT, prothrombin time; INR, international nominal ratio; PTT, partial thromboplastin time; WBC, white blood cells; SOFA, sequential organ failure assessment; GCS, Glasgow Coma Scale; ICU, intensive care unit; LOS, length of stay; bpm, beat per minute.We have obfuscated the number of patients with AIDS out of concern for patient privacy protection.

### MT and all causes in-hospital mortality of IS

**Fig. 2:**
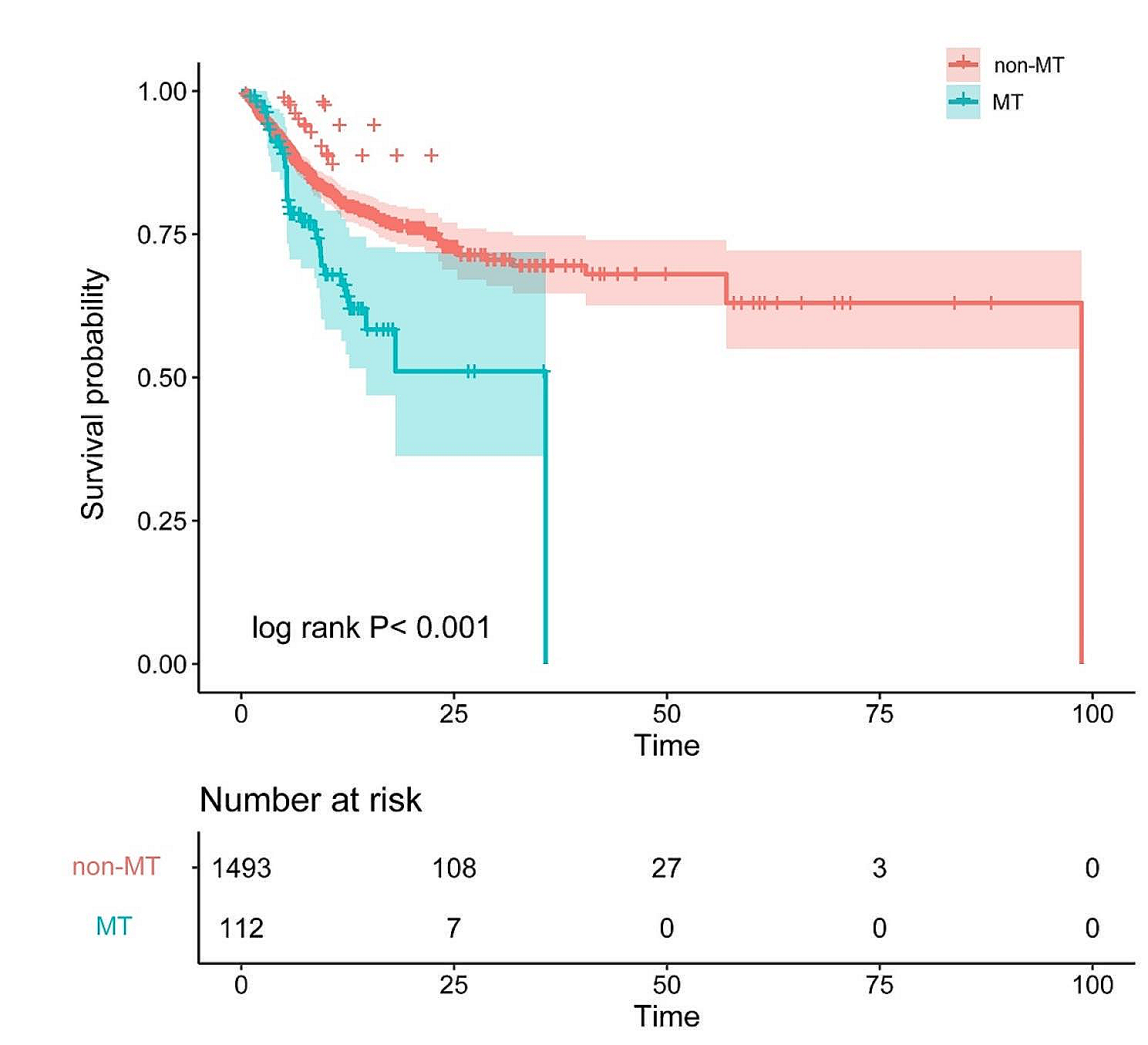
Kaplan-Meier survival curves between MT and non-MT (MT significantly reduced all causes in-hospital mortality in SI patients compared to non-MT)

The Kaplan-Meier analysis plot in Fig. 2 shows the survival curve of patients with MT. The results showed that patients with MT exhibited a significantly lower survival rate during hospitalization compared to those with non-MT (log-rank *P* < 0.001).

In the Cox regression model without adjustments, the presence of MT was associated with elevated in-hospital mortality (HR = 1.905, 95% Cl: 1.320–2.748; *P* < 0.001). After adjusting for confounding factors, three Cox regression models were employed to assess the correlation between MT and in-hospital mortality in patients with IS (Table 2). In Model I, after adjusting for sex, myocardial infarction, diabetes, and weight, MT was associated with an increased risk of in-hospital mortality (HR = 1.842, 95% Cl: 1.2696–2.672; *P* < 0.001). In model II, covariates were further adjusted for vital signs (heart rate and SBP) based on model I, MT patients showed a significantly high in-hospital mortality risk (HR = 1.730, Cl: 1.188–2.520; *P* = 0.004). In model III, the covariates were further adjusted for laboratory data (calcium, sodium, hemoglobin) and SOFA scores based on model II. The analysis revealed a statistically significant elevation in the risk of in-hospital mortality among patients with MT compared to those without MT (HR = 1.844, Cl: 1.255–2.708; *P* = 0.002).

**Table 2:** Association between MT and all causes in-hospital mortality in IS patients

Variable	Crude model		Model I		Model II		Model III	
	HR (95% CI)	*P* value	HR (95% CI)	*P* value	HR (95% CI)	*P* value	HR (95% CI)	*P* value
Non-MT	1.0 (ref)		1.0 (ref)		1.0 (ref)		1.0 (ref)	
MT	1.905 (1.320–2.748)	< 0.001	1.842 (1.2696–2.672)	0.001	1.730 (1.188–2.520)	0.004	1.844 (1.255–2.708)	0.002

Crude model: no adjustment

Model I: adjusted for sex, myocardial infarction, diabetes, and weight.

Model II: adjusted for Model I-added heart rate and SBP.

Model III: adjusted for Model II-added calcium, sodium, hemoglobin, and SOFA.

MT, malignant tumor; IS, ischemic stroke; HR, hazard ratios; CI, confidence interval; SBP, systolic blood pressure; SOFA, Sequential Organ Failure Assessment.

### Subgroup analyses

Among these strata, sex, diabetes, and myocardial infarction were significant clinical risk factors for in-hospital mortality due to IS. Age and SOFA scores are clinical prognostic indicators that have attracted considerable attention. Subgroup analyses were performed separately according to the above variables.

**Fig. 3:**
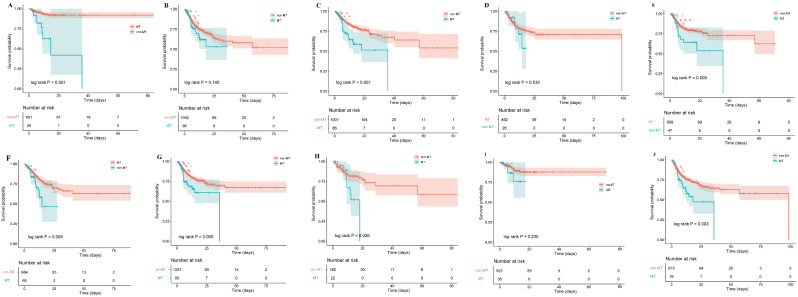
Kaplan-Meier survival analyses of the association between MT and all causes in-hospital mortality in the IS subgroup (A. age ≤ 65, B. age > 65, C. no diabetes, D. diabetes, E. female, F. male, G. no myocardial infarction, H. myocardial infarction, I. SOFA ≤ 2, J. SOFA > 2. MT significantly increased all causes in-hospital mortality in the IS population in the subgroups < 60 years, non-diabetic, female, male, myocardial infarction, non-myocardial infarction, and SOFA > 2)

The Kaplan-Meier analysis plot in Fig. 3 illustrates the survival curves for patients with IS across various subgroup analyses. The results indicate that, among the subgroup aged 65 years or younger, the in-hospital mortality rate of cancer patients is significantly higher than non-MT patients (log-rank *P* < 0.001). Nevertheless, within the subgroup aged over 65 years, while patients with MT showed a lower survival rate during hospitalization than those non-MT, the difference did not reach statistical significance (log-rank *P* = 0.140). In the non-diabetes group, patients with MT exhibited a significantly reduced survival rate during hospitalization compared to those with non-MT (log-rank *P* < 0.001), whereas in the diabetes group, MT had no significant effect on in-hospital mortality. In the SOFA score > 2 group, patients with MT exhibited a significantly reduced survival rate during hospitalization compared to those non-MT (log-rank *P* = 0.003). Additionally, we found that the impact of MT on hospital outcomes was independent of sex and history of myocardial infarction (Table 3).

**Table 3:** Subgroup analysis for MT and all causes in-hospital mortality in IS patients

Variable	Crude model		Model I		Model II	
	HR (95% CI)	*P* value	HR (95% CI)	*P* value	HR (95% CI)	*P* value
Age, years						
< 65	6.576 (3.022–14.31)	< 0.001	4.925 (2.101–11.544)	< 0.001	6.637 (2.690-16.373)	< 0.001
≥ 65	1.378 (0.902–2.105)	0.139	1.303 (0.846–2.005)	0.230	1.422 (0.917–2.204)	0.116
Sex						
Female	2.015 (1.178–3.446)	0.011	1.986 (1.150–3.431)	0.014	2.029 (1.140–3.609)	0.016
Male	1.939 (1.169–3.217)	0.010	1.512 (0.901–2.535)	0.117	1.705 (1.012–2.872)	0.045
Diabetes						
No	2.117 (1.401–3.199)	< 0.001	2.010 (1.317–3.068)	0.001	1.855 (1.197–2.874)	0.006
Yes	1.304 (0.568–2.994)	0.532	1.168 (0.498–2.735)	0.722	1.558 (0.656–3.699)	0.315
Myocardial infarction						
No	1.809 (1.193–2.745)	0.005	1.670 (1.092–2.554)	0.018	1.577 (1.013–2.457)	0.044
Yes	2.430 (1.085–5.442)	0.031	2.406 (1.030–5.620)	0.043	3.037 (1.251–7.370)	0.014
SOFA						
≤ 2	1.862 (0.658–5.269)	0.241	1.737 (0.584–5.164)	0.321	1.451 (0.466–4.525)	0.521
> 2	1.750 (1.175–2.607)	0.006	1.701 (1.137–2.545)	0.010	1.902 (1.261–2.869)	0.002

Crude model: no adjustment

Model I: adjusted for sex, heart rate, SBP, myocardial infarction, diabetes, and weight

Model II: adjusted for Model I-added calcium, sodium, hemoglobin, and SOFA.

MT, malignant tumor; IS, ischemic stroke; HR, hazard ratios; CI, confidence interval; SBP, systolic blood pressure; SOFA, Sequential Organ Failure Assessment

The columns in this figure present the estimated values from independent regression models adjusted for different covariates

### Sensitive analysis

Considering the possible errors associated with multiple interpolation, we finally performed a sensitivity analysis in which only IS patients with complete information were analyzed. It was found that MT was still associated with increased all causes in-hospital mortality among IS patients in the sensitivity analysis (Supplementary Table 2).

## Discussion

In this retrospective study based on a large public database (MIMIC-IV), our findings suggest that the presence of MT could contribute to an elevated risk of all causes in-hospital mortality among patients with IS, even after adjusting for multiple confounders. Patients with comorbid IS and MT exhibited a 1.8-fold higher risk compared to those with IS without cancer. Considering the substantial challenges posed by MT in severe IS patients, clinicians should undertake proactive measures to address this patient population.

MT and IS are two serious health problems currently encountered in the medical field, both of which are characterized by high mortality and morbidity rates. There is a correlation between MT and IS, and many studies have confirmed that patients with MT may face an elevated risk of IS due to factors such as the direct compressive impact of the tumor, vascular infiltration from metastasis, and tumor embolism [17, 18]. Moreover, cancer therapies have been shown to worsen embolic stroke risks, including the acceleration of atherosclerosis induced by radiotherapy and the endothelial damage caused by chemotherapy [17, 19]. On the other hand, stroke may affect the treatment of MT, aggravating their course. The interaction between the two dramatically affects functional status, quality of life, and survival of patients [20]. With the aging population, the emergence of new antitumor drugs, and ongoing improvements in medical care, the survival duration for individuals with MT has been markedly prolonged. Nevertheless, this advancement has concurrently resulted in a heightened risk of stroke for almost all MT survivors over time [7]. The number of patients simultaneously afflicted with MT and IS will continue to rise.

Previous studies have confirmed the correlation between MT and an unfavorable prognosis among patients with IS. Patients with comorbid MT and stroke are significantly younger, have more severe strokes on admission, infarctions occurring across various vascular regions of the brain, occurrences of deep vein thrombosis, coagulation disorders, and a higher risk of all causes mortality than that non-MT [6, 8, 21]. Cancer is associated with long-term mortality in young patients with stroke [22]. Among patients with acute-phase arterial thrombolysis, those with MT often have a worse functional prognosis at 90 days, and higher mortality [23]. Patients with IS due to MT are more likely to be treated in the ICU because of the greater chance of a large infarct, severe symptoms on admission, and the presence of complications. In the present study, we found a significant correlation between MT and the risk of in-hospital mortality in patients with IS in the ICU. The in-hospital mortality rate was significantly high in patients with severe IS in the presence of MT. Additionally, males, had lower body weight, SBP, calcium, sodium, and hemoglobin; and higher heart rate, history of diabetes mellitus, myocardial infarction, and SOFA scores in the MT group were significantly correlated, even after adjusting for relevant confounders using regression modeling.

In this study, an association between MT and increased risk of in-hospital death in patients with IS was observed in the age, sex, history of diabetes mellitus, history of myocardial infarction, and SOFA score subgroups. Similar conclusions were reached in the sex and history of myocardial infarction subgroups but not in the age, history of diabetes mellitus, and SOFA score subgroups. First, malignancy was associated with an increased risk of in-hospital mortality in patients aged < 65 years with cerebral embolism, whereas a similar association was not observed in those aged > 65 years. This may be because the pathogenesis of tumor-associated IS differs between older and younger patients. In general, elderly patients are more likely to have underlying diseases such as atherosclerosis, hypertension, hyperlipidemia, and diabetes mellitus, which increase the risk of cerebrovascular injury and obstruction. Older patients were more likely to have IS of large or small arteries, whereas younger patients were more likely to have IS of cardiac or other origins. Cardiac or cryptogenic cerebral infarcts, which present as multiple cerebral infarcts, are more common and associated with higher D-dimer levels [24]. Elderly patients with IS frequently present with multiple underlying diseases, including hypertension, diabetes, and cardiac disease. These comorbidities contribute to the heightened severity of IS and an increased risk of complications and may mask the influence of malignancy on in-hospital mortality. Secondly, malignancy was associated with an increased risk of in-hospital mortality in patients with IS without a history of diabetes. We believe this may also be related to different stroke types. Previous research indicates that stroke types in diabetic patients are more often characterized by atherosclerotic thrombotic stroke and lacunar infarctions [25]. In contrast, among non-diabetic patients, there is a higher incidence of cerebral embolism, and these patients often have a poorer prognosis, masking the effect of malignancy on in-hospital mortality. Finally, we observed that in the subgroup with a SOFA score > 2, malignancy was associated with an increased in-hospital mortality rate among patients with IS. However, no such association was found in the subgroup with a SOFA score ≤ 2. MT may exacerbate IS in patients with severe organ dysfunction, thereby affecting prognosis.

The primary strength of this study lies in our finding of cancer as a significant independent risk factor for heightened mortality in critically ill patients with IS in a US cohort. Nonetheless, the study has a few limitations. Firstly, due to its retrospective design, establishing definitive causality is challenging. Despite conducting multivariable adjustments and subgroup analyses, residual confounding factors may have influenced clinical outcomes. Variables such as time of stroke, IS subtypes, National Institutes of Health Stroke Scale (NIHSS), and cause of death were inaccessible in this database [26]. Secondly, the analysis did not consider the type and stage of cancer, whether it was metastatic, or the specific treatment received. Additionally, given the database’s nature, only the short-term mortality risk of patients could be investigated. Therefore, further research is imperative to ascertain the long-term prognostic impact of cancer in critically ill patients with IS.

## Conclusions

Among patients with IS, the all causes in-hospital mortality risk in those with MT was higher than in non-MT patients. This finding can assist clinicians in more accurately assessing prognosis and making informed treatment decisions.

## Data Availability

No datasets were generated or analysed during the current study.

## Abbreviations

AIDS: Acquired immunodeficiency syndrome
BUN: Blood urea nitrogen
CI: Confidence intervals
DBP: Diastolic blood pressure
GCS: Glasgow Coma Scale
HR: Hazard ratio
ICU: Intensive care unit
INR: International nominal ratio
IS: Ischaemic stroke
LOS: Length of stay
MBP: Mean blood pressure
MIMIC-IV: Medical Information Mart for Intensive Care IV
PT: Prothrombin time
PTT: Partial thromboplastin time
RR: Respiratory rate
SBP: Systolic blood pressure
SD: Standard deviation
SOFA: Sequential organ failure assessment
SPO2: Pulse oximetry-derived oxygen saturation
T: Body temperature
WBC: White blood cells
